# CystoDS: a multiclass endoscopy image dataset for artificial intelligence-assisted bladder cancer detection

**DOI:** 10.1038/s41597-026-06887-z

**Published:** 2026-02-26

**Authors:** Timothy J. Lee, Liang Qiu, Jin Long, Kathleen E. Mach, Dylan Peterson, Qingsong Yao, Eugene Shkolyar, Md Tauhidul Islam, Lei Xing, Joseph C. Liao

**Affiliations:** 1 Department of Urology, Stanford University School of Medicine, Stanford, USA; 2 Veterans Affairs Palo Alto Health Care System, Livermore, USA; 3 Center for Artificial Intelligence in Medicine & Imaging, Stanford University School of Medicine, Stanford, USA; 4 Department of Radiation Oncology, Stanford University School of Medicine, Stanford, USA

**Keywords:** Bladder cancer, Cancer imaging

## Abstract

Cystoscopy is a common endoscopic procedure used for the visual inspection of the lower urinary tract, particularly for the detection and surveillance of bladder cancer. Artificial intelligence (AI) strategies could help address the recognized shortcomings of cystoscopy by identifying malignant and non-malignant regions of interest (ROIs) and providing real-time clinical decision support for biopsy and surgical resection. However, curating a dataset for training AI models is challenging and time-time consuming. We present CystoDS, a high-quality bladder imaging dataset derived from standard white light cystoscopy that is ready for AI applications for detection of bladder cancer and cancer-mimicking benign lesions. This dataset includes 8,067 images from 160 patients labelled with five classes and 22 subclasses, along with segmentation data for 768 of the images. We detail the methods used for image acquisition, the structure of the dataset, and our technical validation process to demonstrate the AI-readiness of the data.

## Background & Summary

Bladder cancer is the 6^th^ most commonly diagnosed malignancy in the United States and disproportionately affects males, with 63,070 new cases in males compared to 20,120 new cases in females in 2024^1^. White light cystoscopy is the standard endoscopic method for visual identification of suspected bladder cancer and other regions of interest (ROI) in the bladder^2^. In a clinic setting, a flexible cystoscope is inserted through the urethra into the bladder to visualize the mucosa and identify any abnormalities. If tumors or ROIs are detected, an endoscopic surgical procedure called transurethral resection of bladder tumor (TURBT) is performed in the operating room to treat the tumor and establish a pathologic diagnosis^3^.

Standard white light cystoscopy has several recognized shortcomings including difficulty in differentiating cancer from non-cancerous ROIs,^4,5^ detecting non-papillary carcinoma *in situ* (CIS) that lacks visually distinct borders^6–8^, enumerating multifocal tumors, and ensuring complete tumor resection^9^. Bladder cancer has the highest lifetime treatment costs of any cancer^10^, highlighting the need to address these shortcomings. Efforts in this space include enhanced cystoscopy technologies such as blue light cystoscopy, which requires instillation of the optical imaging agent hexaminolevulinate^11^. Currently, blue light cystoscopy is recommended by recognized guidelines, if available, to increase tumor detection^12^.

Artificial intelligence (AI) and deep learning have the potential to enhance visualization, enabling surgeons to better identify, evaluate, and treat bladder cancer. We previously reported CystoNet, an AI model that accurately detects bladder tumors during cystoscopy^13,14^. While this model demonstrated promising performance for papillary tumors, to create more comprehensive and accurate AI tools, a large dataset of quality images following FAIR principles (Findable, Accessible, Interoperable, Reusable) is required for model training and testing^15^. As new deep learning techniques become available, having AI-ready datasets with accurate, complete, and consistent data becomes imperative for model training and validation^16^. While several large endoscopic imaging datasets exist for other organ systems, such as the gastrointestinal tract^17–19^, large cystoscopy imaging datasets for training AI models are lacking. One bladder endoscopy dataset used to train a classification model has been published^20^. Another small cystoscopy imaging atlas derived from a textbook was published, but it is limited due the small size of the dataset and suboptimal image quality^21^.

In this study, we present CystoDS^22^, an accessible cystoscopy image dataset containing 8,067 labelled images from 160 patients. These labelled images include pathologically confirmed malignant and non-malignant ROIs, anatomical landmarks of the bladder, foreign bodies, and normal bladder mucosa. We performed technical validation using our labelled dataset to demonstrate the feasibility and potential usability of CystoDS for AI model training and validation. The benchmark performance of CystoDS was validated using publicly available deep learning image classification models towards developing AI-driven solutions to enhance endoscopic bladder tumor detection and resection.

## Methods

**Fig. 1:**
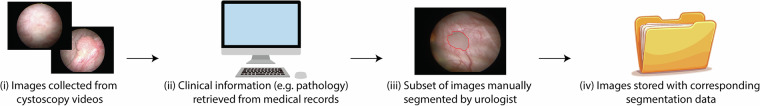
CystoDS imaging data acquisition and storage. A total of 8,067 images were captured from cystoscopy videos. Images were then labelled with relevant clinical and pathological information retrieved from medical records. 768 images containing regions of interest were additionally segmented and reviewed by expert urologists and then stored with corresponding segmentation data in JSON format.

With Institutional Review Board (IRB #29838 and #36085) approval, human subjects (n = 160) undergoing cystoscopy and TURBT were consented and enrolled from 2016 to 2022 at the Veterans Affairs Palo Alto Health Care System (VAPAHCS). Patients consented to recording of standard of care cystoscopies to be uploaded to a secure database as de-identified files that can be shared electronically. Informed consent was obtained at the time of cystoscopy by trained research personnel. Only the protocol director and his trained research personnel have full access to patient data. Table 1 shows patient demographics and diversity of images and ROIs. Video and image data were acquired at the time of TURBT (n = 200, KARL STORZ Endoskope, Tuttlingen, Germany) in the operating room or flexible cystoscopy (n = 8, Olympus Corporation, Tokyo, Japan) in the clinic. Additional still images were captured from cystoscopy videos using built-in MacOS screenshotting software and FFmpeg, an open-source video processing software (ffmpeg.org). A schematic of data acquisition and storage is shown in Fig. 1.

**Table 1:** Patient demographics and diversity of images and ROIs.

n		160
Age, median (IQR)		78 (75, 83)
Race, n (%)	White	130 (81.2)
	Non-white	30 (18.8)
Ethnicity, n (%)	Hispanic or Latino	15 (9.6)
	Not Hispanic or Latino	138 (87.9)
	Unknown or not reported	4 (2.5)
Male, n (%)		159 (99.4)
Images per patient, median (IQR)		8 (4, 45)
ROIs per patient, median (IQR)		1 (1, 2)

Participants were enrolled at a Veterans Affairs medical center, where majority of patients are males. Images per patient includes all types of images (malignant, non-malignant, anatomical landmarks, foreign bodies, and normal mucosa). ROIs per patient includes both malignant and non-malignant.

All images (n = 8,067) were labelled after clinical and pathological diagnoses were established. The labelling process involves renaming image file names with clinical information following a standardized protocol^23^. This clinical information served as the basis of the class and subclass determination for each image. File names of images included in CystoDS were changed to a random 8-digit alphanumeric sequence to protect patient privacy and only relevant de-identified clinical data were stored in the accompanying cystods.csv file.

ROIs in select images (n = 768, 9.6% of total images) were segmented by an expert urologist using LabelMe. Segmentation consisted of outlining ROIs considered suspicious enough to warrant a biopsy and/or surgical resection for pathological diagnosis. Segmented images were then reviewed by another expert urologist for quality control. Data of verified segmentations were stored in JSON files.

## Data Records

The complete CystoDS dataset^22^, including images, segmentation data, and metadata, is available from Open Science Framework (OSF) at https://osf.io/xvdhy/. CystoDS comprises three main components: the “image” folder, “segmentation” folder, and “cystods.csv” file. The “image” folder contains all images included in the dataset (n = 8,067) in PNG format, with resolutions ranging from 252 to 5120 pixels in width and 209 to 2880 pixels in height. The median file size is 95 kilobytes (KB), with a range of 44 to 8787 KB. The “segmentations” folder contains JSON files that store segmentation data for the segmented images (n = 768). Similar to other published endoscopy datasets, not all images in CystoDS have associated segmentation data (Table 2); however, all images in CystoDS have a class label. CystoDS has a comparable number of segmented and labelled images to existing endoscopy datasets such as HyperKvasir^17,24^ (n = 10,662 labelled images; 1,000 segmented images), PolypGen^18,25^ (n = 8,037 labelled images; 1,537 segmented images), and GastroVision^19,26^ (n = 8,000 labelled images; 0 segmented images). Compared to the bladder endoscopy dataset published by Lazo *et al*.^20,27^ (n = 1,754 labelled images; 0 segmented images; 23 patients), CystoDS offers images from a greater number of patients (n = 160) and segmentation data for a subset of the dataset. Additionally, CystoDS contains a higher percentage (79%) of normal mucosa images. This skew towards normal mucosa images reflects the reality of bladder endoscopy where typically more normal mucosa is visualized compared to abnormal. Users of our dataset can address the potential bias that comes with class imbalance by randomly selecting a subset of normal mucosa images during training.

**Table 2:** Publicly available gastrointestinal and bladder endoscopy datasets compared to CystoDS.

Dataset	Labelled Images	Segmented Images	Patients
HyperKvasir^17,24^	10,662	1,000	N/A
PolypGen^18,25^	8,037	1,537	300+
GastroVision^19,26^	8,000	0	N/A
Lazo *et al*.^20,27^	1,754	0	23
CystoDS^22^	8,067	768	160

HyperKvasir, PolypGen and GastroVision are AI-ready endoscopy image datasets available for the gastrointestinal tract, while Lazo *et al*. is an AI ready bladder image dataset. Labelled images include images that have been assigned a class. Segmented images include images that were segmented by a urologist. There is overlap between labelled and segmented images. N/A = not applicable.

**Fig. 2:**
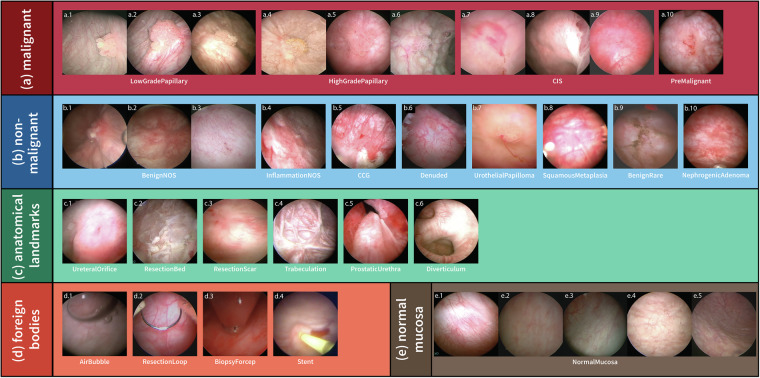
CystoDS classes, subclasses and representative images. A total of 8067 images from 160 patients were divided into five classes: (**a**) malignant; (**b**) non-malignant; (**c**) anatomical landmarks; (**d**) foreign bodies; and (**e**) normal mucosa. Malignant includes pathologically confirmed papillary urothelial carcinoma, carcinoma *in situ* (CIS), and a pre-malignant urothelial tumor. Non-malignant are cancer-mimicking in appearance but confirmed pathologically to be benign. Anatomical landmarks are structures (e.g. ureteral orifice) or features within the bladder as determined by experienced urologists. Foreign bodies include surgical instruments and other findings that are not normally present in the bladder. Normal mucosa is visually normal-appearing bladder tissue. Abbreviations: NOS (not otherwise specified), CCG (cystitis cystica and glandularis).

**Fig. 3:**
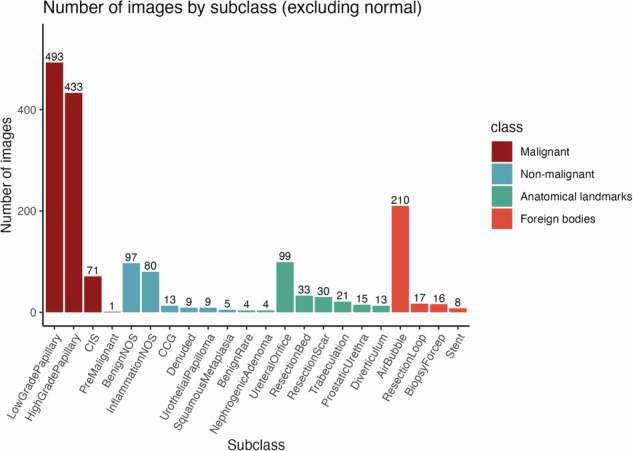
CystoDS images by class and subclass. The 22 subclasses are grouped into five classes: Malignant (n = 998), Non-malignant (n = 221), Anatomical landmarks (n = 211), Foreign bodies (n = 251). Normal mucosa images (n = 6,386) are not shown.

**Fig. 4:**
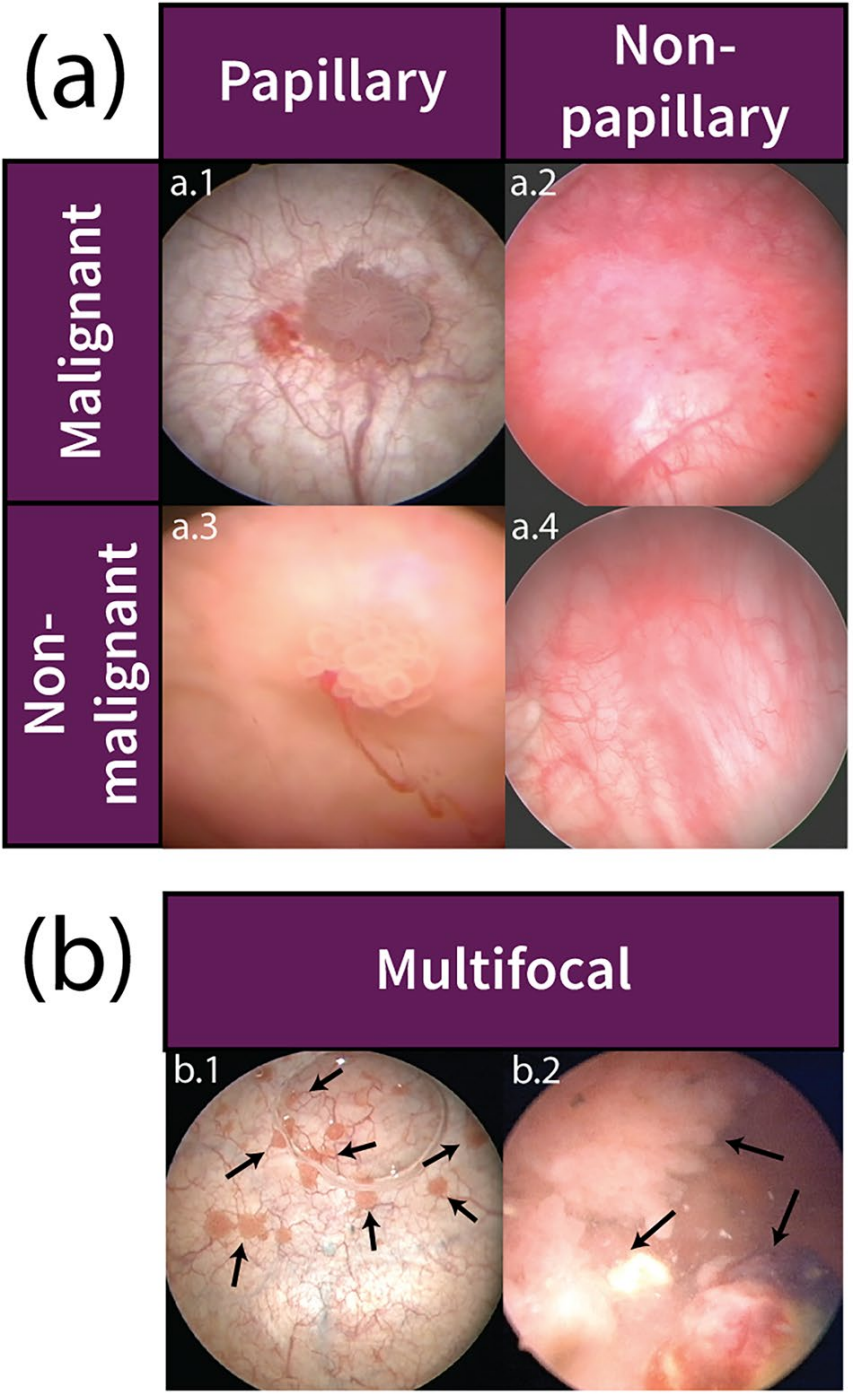
Representative images of bladder tumor morphology and multifocality. Morphology and multifocality are additional labels included in the metadata for the dataset. (**a**) ROIs that appear papillary (n = 875) or non-papillary (n = 344) can be either malignant or non-malignant. The malignant LowGradePapillary ROI depicted in a.1 appear morphologically similar to the non-malignant UrothelialPapilloma shown in a.3. Similarly, the CIS in a.2 and BenignNOS are difficult to differentiate. (**b**) Images labelled as multifocal (n = 100) contain multiple malignant tumors within a single image. This creates a challenge in enumerating tumors within a bladder.

The file, cystods.csv, contains the metadata for all 8,067 images including variables such as filename, participant identification number (pid), visit, ROI, multifocal, bladder cancer status (bca), class, subclass, subclass2, stage, morphology, modality, and JSON. The “filename” variable is a randomized image file name that was generated using the ids package in R to deidentify any protected health information (PHI) in the original file name. Every participant was assigned a random patient identification number (pid) after obtaining informed consent and prior to data collection. Due to the recurrent nature of bladder cancer, participants often undergo multiple cystoscopies for disease surveillance and therefore contributed to the dataset multiple times. Specifically, there are images from one (n = 83 patients) or two (n = 57) visits, with some contributing images from three (n = 14), four (n = 3), five (n = 2), and seven (n = 1) visits. Visits were numbered sequentially for each individual and stored in the “visit” variable. Within any given visit, multiple ROIs may be identified, and multiple images of the same ROI from different perspective can be generated. All images are assigned a “class” and “subclass”: the five classes – *malignant, non-malignant, anatomical landmarks, foreign bodies*, and *normal mucosa* are further defined into 22 subclasses (Figs. 2, 3). The “ROI” variable denotes the identification number for the given image, with different numbers represent different ROIs or identify the same ROI from different perspectives. In cases where enumeration of ROIs was not feasible, or multiple ROIs were sent for pathology as one specimen, images were labelled “multifocal” instead of a number for their ROI variable (Fig. 4b). Additionally, the “multifocal” variable is assigned “2–7” or “8+” as an estimate of the number of tumors associated with the multifocality. The “bca” variable is set to 1 for images containing bladder cancer and 0 when no cancer is present in the image.

- *Malignant* subclasses (n = 4): *LowGradePapillary*, *HighGradePapillary*, *CIS*, and *PreMalignant* are subclasses based on guidelines for non-muscle invasive bladder cancer (NMIBC)^28^. *PreMalignant* includes urothelial tumors that are not assigned a grade or stage such as urothelial proliferation of undetermined malignant potential (UPUMP) and papillary urothelial neoplasms of low malignant potential (PUNLMP)^29^.
- *Non-malignant* subclasses (n = 8): *BenignNOS* (benign, not otherwise specified), *InflammationNOS* (inflammation, not otherwise specified), *CCG* (cystitis cystica and glandularis), *Denuded* (denuded urothelium), *UrothelialPapilloma, SquamousMetaplasia, NephrogenicAdenoma*, and *BenignRare* are subclasses determined through internal discussion between expert urologists and pathologists to consolidate various benign ROIs that can mimic bladder cancer on cystoscopy.
- *BenignNOS* includes pathological findings such as reactive changes, urothelial mucosa, atypia, and dysplasia. *InflammationNOS* includes the various non-specific diagnoses of cystitis, while *CCG* is a more specific diagnosis associated with chronic inflammation. *BenignRare* includes rare cystoscopic findings such as malakoplakia and melanosis.
- *Anatomical landmarks* subclasses (n = 6): *UreteralOrifice, ResectionBed, ResectionScar, ProstaticUrethra*, *Trabeculation*, and *Diverticulum* are common anatomical landmarks observed in patients at risk for bladder cancer^30^.
- *Foreign bodies* subclasses (n = 4): *AirBubble*, *ResectionLoop*, *BiopsyForcep*, and *Stent* are common cystoscopy findings and tools.
- *Normal mucosa*: Images of visually normal-appearing bladder tissue are assigned this class and *NA* (not applicable) for subclass.

**Fig. 5:**
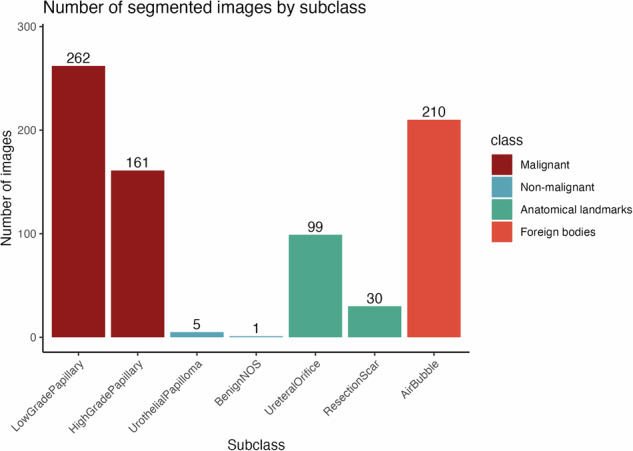
768 segmented images in CystoDS by class and subclass. Segmented images are images with a corresponding segmentation mask that was created by a urologist and reviewed by a second urologist. Normal mucosa images were not segmented.

A subset of cases contains ROIs with complex pathology. Twelve ROIs are pathologically low grade with focal high grade papillary urothelial carcinoma. In these cases, the images were assigned class *Malignant*, subclass *LowGradePapillary*, and *HighGradePapillary* for “subclass2”. In a four cases CIS was present in addition to papillary urothelial carcinoma. These images were assigned class *Malignant*, subclass *HighGradePapillary*, and *CIS* for subclass2. Bladder cancer pathology also includes local stage information (T0, Ta, Tis, T1, T2) for depth of tumor involvement. These data are stored in the “stage” variable. Bladder cancer pathology is defined by both the grade and stage. The 998 images of malignant ROIs in the dataset fall into five combinations of grade and stage: LG (low grade) Ta (n = 491), LG T1 (n = 2), HG (high grade) Ta (n = 210), HG T1 (n = 170), and HG T2 (n = 53). Images that do not contain malignant ROIs with stage information are assigned *NA* for stage. “Morphology” refers to the physical appearance of an ROI. Malignant and non-malignant ROIs are assigned either *papillary* or *non-papillary* morphology (Fig. 4). In some cases, blue light cystoscopy was used in addition to white light cystoscopy. Images are assigned either BLC (n = 450) or WLC (n = 7,617) respectively under “modality”. If an image has an associated segmentation file, “JSON” is set to 1; otherwise, it is set to 0 (Fig. 5).

## Technical Validation

We performed technical validation of the CystoDS dataset using publicly available deep learning models to demonstrate its reliability and suitability for bladder ROI classification tasks.

### Experimental setup

We trained four widely used deep learning models for medical image classification to establish benchmark performance on the dataset. These models were selected to evaluate both standard convolutional neural network (CNN) architectures and advanced transformer-based designs, providing a comprehensive basis for comparison. ResNet^31^ is a CNN known for its straightforward design and robust feature extraction capabilities. ResNeXt^32^ extends ResNet with a grouped convolution strategy, balancing efficiency and complexity to handle diverse ROI types. HRNet^33^ preserves high-resolution representations throughout the network, making it particularly effective for capturing fine-grained details and spatial accuracy in medical imaging tasks. Swin-Transformer^34^, a state-of-the-art vision transformer, utilizes a hierarchical self-attention mechanism with shifted windows, offering superior performance by modelling both local and global features.

To enable training, validation, and internal testing for classification of regions of interest (ROIs), we randomized the CystoDS dataset into three subsets following a 70:15:15 split. Randomization was performed at the patient level ensuring that no patient’s data appeared in more than one subset. The final data splits include a training set of 1,772 images from 128 patients for model training, a validation set of 226 images from 15 patients for hyperparameter tuning and early stopping, and a test set of 219 images from 17 patients for final model evaluation. Furthermore, we simplified the classification task for our model by grouping image classes into two categories: ROI and non-ROI. The ROI group (n = 1,215) included *malignant* images (n = 994) and *non-malignant* images (n = 221). The non-ROI group (n = 1,002) consisted of *anatomical landmarks* (n = 421), *foreign bodies* (n = 41), and a randomly selected subset of n*ormal mucosa* (n = 540) from 6,386 available images. Since *normal mucosa* was the largest class, limiting its inclusion to 540 images (~10%) helped balance the dataset and minimize potential model bias.

To further assess generalizability of CystoDS, we performed external validation by applying the models trained on CystoDS images to the independent bladder endoscopy dataset published by Lazo *et al*.^20,27^, without any fine-tuning.

### Validation results

To evaluate ROI classification performance of the models with the CystoDS dataset, standard metrics including sensitivity, specificity, accuracy, precision, and F-1 score were determined.

Table 3 shows the variation in standard metric across the different deep learning models for internal and external validation. Trained on the CystoDS dataset, Swin-Transformer achieved the highest performance across all metrics, with an accuracy of 0.831 and 0.873 in internal and external validation, respectively and F1-scores of 0.856 and 0.862 in internal and external validation, respectively. This superior performance highlights the potential of hierarchical attention mechanisms to address the diverse characteristics of CystoDS, positioning it as a promising avenue for future research. With the external validation set the models exhibited varying levels of cross-dataset performance, with some demonstrating strong generalizability.

**Table 3:** Results for four deep learning classification models trained on CystoDS, tested internally on the CystoDS test set and externally on the dataset published by Lazo *et al*., providing an evaluation of the generalizability and usability of CystoDS.

Model	Sensitivity	Specificity	Accuracy	Precision	F1-Score
**Internal Test (CystoDS**^22^)					
ResNet^31^	0.692	0.787	0.731	0.826	0.753
ResNeXt^32^	0.754	0.787	0.767	0.838	0.794
HRNet^33^	0.692	0.910	0.781	0.918	0.789
Swin-Transformer^34^	0.846	0.809	0.831	0.866	0.856
**External Test (Lazo** ***et al***.^20,27^)					
ResNet^31^	0.664	0.746	0.708	0.694	0.678
ResNeXt^32^	0.506	0.779	0.584	0.851	0.634
HRNet^33^	0.555	0.575	0.561	0.764	0.643
Swin-Transformer^34^	0.853	0.890	0.873	0.870	0.862

The variability in model performance further emphasizes the importance of tailoring model selection to specific clinical objectives. Moreover, the dataset’s high-quality segmentations and detailed categorization enable researchers to evaluate model performance across a wide range of ROIs and non-ROIs, driving advancements in diagnostic accuracy and clinical utility.

## Data Availability

The complete CystoDS dataset^22^, including images, segmentation data, and metadata, is available from Open Science Framework (OSF) at 10.17605/OSF.IO/XVDHY.

## Code availability

The code used to test the dataset is available on GitHub at https://github.com/liaolabsu/CystoDS.
